# PURPOSE IN LIFE, STRESS REACTIVITY, AND COGNITIVE AGING: A LONGITUDINAL INVESTIGATION

**DOI:** 10.1093/geroni/igac059.2168

**Published:** 2022-12-20

**Authors:** Niccole Nelson, Cindy Bergeman

**Affiliations:** University of Notre Dame, Notre Dame, Indiana, United States; University of Notre Dame, Notre Dame, Indiana, United States

## Abstract

This study examined (1) the time-varying relationship between purpose in life and perceived stress reactivity, (2) the trajectory of perceived stress reactivity as it relates to both within- and between-person purpose in life, as well as (3) the predictive utility of perceived stress reactivity and its rate of change on cognitive ability and allostatic load. The sample comprised 933 participants from the Notre Dame Study of Health & Well-being, a 10-year study of annual questionnaire packets and biennial daily diary bursts. Analyses included three-level multilevel models from which random effects were extracted and used to predict allostatic load and cognitive ability. Results indicated that individuals were affectively reactive to perceived stress, and that perceived stress reactivity declined over time. Considering the effects of purpose in life on these processes, there were two cross-level interaction effects indicating (1) more purposeful individuals were less stress reactive than less purposeful individuals, and (2) more purposeful individuals declined less in negative affect over time than less purposeful individuals. There was also preliminary evidence for a within-person interaction effect between yearly purpose in life and daily affective reactivity such that when individuals felt particularly purposeful, they also tended to be less stress reactive. Finally, higher perceived stress reactivity, as well as less decline in this construct, was predictive of better cognitive ability. These findings indicate purpose in life buffers against environmental and maturational effects on negative affect, and that perceived stress reactivity may indicate a different, more adaptive process than affective reactivity to experienced stressors.

